# Validity and reliability study of clinician attitude towards rural health extension program in Ethiopia: exploratory and confirmatory factor analysis

**DOI:** 10.1186/s12913-022-08470-9

**Published:** 2022-08-25

**Authors:** Merhawi Gebremedhin, Esie Gebrewahd, Lauryn K. Stafford

**Affiliations:** 1 School of Public Health, Haramaya University, Harar, Ethiopia; 2 School of Geography and Environmental Studies, Haramaya University, Harar, Ethiopia; 3 Institute for Health Metrics and Evaluation, University of Washington, Seattle, USA

**Keywords:** Attitude, Clinician, Ethiopia, Health extension program

## Abstract

**Background:**

The Ethiopian health extension program (HEP) is an innovative community-based strategy aimed at disease prevention and health promotion. While health extension workers (HEWs) are its front-line workers, the involvement of clinicians remains an integral part. The goals of this study were to: (1) assess the correlation of clinician attitude with predictors and (2) assess the reliability and validity of the survey instrument.

**Methods:**

A cross-sectional study design was utilized to collect data from a sample of 1239 clinicians using 28 items of attitude questions. Exploratory factor analysis (EFA) was applied to create the latent variables. Oblique Promax type rotation with factor loading (> 0.5) was used. Cronbach’s alpha was used to assess reliability, with a level of > 0.7 suggesting good reliability. Confirmatory factor analysis (CFA) was undertaken, with the values of Root Mean Square Error Administration (RMSEA) < 0.08, Standardized Root Mean Square Residual (SRMR) < 0.05, comparative fit index (CFI) 0.9–0.95, and Tucker-Lewis index (TLI) 0.9–0.95 suggesting acceptable model fit. A linear regression analysis was conducted.

**Results:**

EFA produced two latent variables which explained 93.2% of the total variance. The latent variables were labeled as perceived attitude towards the skill of HEWs (F1), and perceived attitude towards the impact of HEP (F2). Internal reliability for the 28 items was reported with a Cronbach’s alpha of 0.94, and for F1 and F2 it was 0.91 and 0.90, respectively. CFA was done and RMSEA was reported at 0.04, SRMR was 0.03, and CFI and TLI were each 0.97. The value of clinician attitude increased by 3.5, 95% CI (1.5, 5.3), *P*-value < 0.001 for those who have been exposed to the HEP program than non-exposed. Similarly, clinician attitude was lower for degree holders compared to those with diplomas by − 2.7, 95% CI (− 4.4, − 0.94), *P*-value < 0.002.

**Conclusion:**

Clinician attitude increased as exposure to HEP increased. Clinician attitude towards HEP has two latent variables. Furthermore, the assessment tool demonstrated good reliability and validity. In conclusion, it is worthy valued for clinicians to receive orientation about HEP, and researchers and program evaluators can use this assessment tool.

## Background information

Over the past two decades, Ethiopia has achieved a remarkable improvement in family and community health [1]. The Health Extension Program (HEP), which is considered a flagship innovative intervention, has contributed significantly to scaling-up primary health care services [1, 2]. HEP was launched in 2003 [3, 4] to comply with various international declarations, as well as to ensure fair distribution of and accessibility to basic health services for citizens [5]. Its major objective has been to distribute health services fairly to the Ethiopian people through family and community-centered health promotion and disease prevention [6]. To achieve these objectives, 18 health packages structured into four components were designed [3].

The program is primarily executed by female paid staff, known as health extension workers (HEWs) [4, 7–9]. So far, about 39,000 HEWs have been deployed in 17,000 health posts (HP), assuming two HEWs per HP. [3] Many stakeholders, including all levels of the health sector, teaching institutions, political leaders, and international partners, have been involved in its implementation [5]. In the beginning, the respective Woreda health offices were responsible for providing technical and administrative support for the program. However, following the shared experience from the agricultural extension program, supervisors educated in environmental health were assigned to program support in 2008. These graduates do not support clinical services; therefore, the support, follow-up, and evaluation of the program has shifted to health centres (HC) and hospital technical staff, who are collectively called clinicians [9]. These include nurses, health officers (HO), midwives, integrated surgical and obstetricians (ISO), and medical doctors (MD).

Clinicians are responsible for preparing plans, giving technical and administrative support to health posts (HP), data gathering and analysis, providing on-job training for HEWs, sharing best experiences among HPs in the catchment, helping HPs through outreach, assigning HC staff to HPs, evaluating HP performance, and sending reports to the Woreda health office [10]. Hospital staff are also responsible for supporting the program through the provision of training, preparing review meetings, field supervision, and other related activities [11]. Despite the fact that clinicians are tasked with supporting the program, their attitudes towards HEP implementation efforts have not yet been assessed in Ethiopia. We believe that clinician attitude has a crucial role in the continuity and successful implementation of the HEP program, with a major role in leadership and HEWs training. Therefore, the purpose of this study was: (1) to examine the reliability and validity of the clinician attitude measurement tool in order to apply the tool in future studies and (2) to identify factors correlated with clinician attitude.

## Methodology

### Study setting and design

Ethiopia has 4000 HCs, 400 public hospitals, and nearly 17,000 HPs [12]. After several reforms, the country now has three health system tiers, one of which is the primary health care unit (PHCU), consisting of primary hospitals, HCs, and HPs that serve the majority of the population. The second and third tiers are general and referral hospitals, where most curative services are provided [12, 13]. The HEP is not a standalone program; rather, it is integrated into the public health system. According to this structure, HEP is expected to receive guidance from HCs (there are an average of five HPs per HC), primary hospitals, and district health administrations. HC is staffed by nurses, midwives, and HOs; PH is staffed by MD and ISO in addition to the staff mentioned in HC. GH and RH are staffed by nurses, midwives, ISO, and MD. HPs are staffed by two HEWs and serve an average of 3000–5000 people [14].

### Definition, study design & procedures

A clinician is operationally defined as a health provider with the qualification of nurse (diploma and degree), or midwifery (diploma and degree), or HO, or ISO (master), or MD (general practitioner, pediatrician, or gynecologist-obstetrician) that is working in a public facility (HC or hospital) with at least one or more service years in the facility.

A cross-sectional study design was used to gather data from clinicians working in public facilities (HCs and hospitals). A decision was made to use positive attitude proportion data from a pilot study result because a comparative study had not been conducted previously. The results of the pilot study showed that the proportion of clinicians with favorable attitudes (composite score value > = 80% based on Bloom’s classification of attitude) [15] was reported at 37.5%. Taking this into account, sample size was calculated using a single proportion formula with a 95% confidence interval (CI), marginal error of 0.04%, design effect of 2, and non-response rate of 10%. This gave a final sample size of 1239.

This survey was part of the second-round survey under the umbrella of “HEP assessment,” in which data was collected in two rounds. In the first round, 169 HCs from 64 rural Woredas from nine regions were selected randomly for facility assessment. An urban HEP assessment was conducted in the second round and 45 HCs (38 from AA & 7 from DD) were included in the survey. All HCs (214) were included in this survey. Hospitals for assessment were selected from the national list; 15 primary hospitals (PH), 11 general hospitals (GH), and 10 referral hospitals (RH) were surveyed randomly. The number and type of clinicians to be interviewed per facility were determined by taking standard staff deployment into consideration [12]. Five clinicians, including two nurses (degree and diploma), two midwives (degree and diploma), and one HO, were interviewed from each HC. This number increased to seven clinicians in the case of PHs, where one GP and one ISO were also interviewed. Two nurses (diploma and degree), two midwives (diploma and degree), one ISO, one GP, one Gyne-Obs, and one pediatrician were interviewed from general and referral hospitals. Generally, we utilized systematic random sampling to choose clinicians and simple random sampling to select health facilities. The Kish-method was used to select clinicians within the facility [16]. Once the facility was selected, the data collector visited the maternal and child health (MCH) department, due to the assumption that clinicians in the MCH department have a direct role in leading the HEP program. The data collector first asked the department head about the number of providers available at the time of data collection. For example, if there was only one HO in a given department, the data collector would interview him directly. However, if there were more than two HOs, the data collector recorded their names in alphabetical order and chose one HO from the kish-gride. The same procedure was used for the other qualifications.

### Data collection

In order to collect the relevant data, a survey tool was initially developed based on national HEP guidelines and various literatures. A structured questionnaire was then rearranged and commented on for face validation by experts working in FMoH and social educators, such as psychologists. The final version of the questionnaire was translated from English to Amharic and pretested among 51 clinicians (4.1% of the sample) working in three different non-sampled Woredas. Data collectors and supervisors who are degree holders in health sciences and have extensive work experience were recruited and deployed to collect data. A face-to-face interview with an open data kit (ODK) was used to collect data, as data collectors were well trained on its usage and application. Immediately after the completion of the interview, the data was submitted to the central server data storage. The data manager monitored the incoming data centrally and notified field staff on the spot if any potential errors were happening throughout the data collection period.

The survey tool included 28 items with attitude questions. The questions were written with positive statements, and responses were recorded using the five Likert-scale method coded zero to four, where zero represents “strongly disagree” and four represents “strongly agree.” A composite score value was generated for the 28 items, which gave a 0–112 total score.

### Data description

Data were downloaded in CSV format from the central server and then exported to STATA version 14 for detailed analysis. Descriptive data was used to check for missing values and proportions were used to describe the study variables. Data cleaning and recoding were done accordingly. Outliers were checked using graphs, and corrections were made using the winsor2 method. The response rates of all items were also checked. The means and medians of each item were then summarized to determine the item distribution. A composite score value was generated for the 28 items, giving a range from 0 to 112. We assessed the floor and ceiling effects from the composite score value; if > = 15% of clinicians scored the lowest value, floor effect (0/112), or the best value, ceiling effect (112/112). In order to evaluate the validity and reliability of the survey instrument on clinician’s attitudes towards HEP, internal consistency and factorial validity were conducted following EFA and CFA, respectively. Multivariate regression methods were used on selected predictors.

### Exploratory factor analysis

Exploratory factor analysis (EFA) was applied to determine latent variables. Three basic steps, including (i) sample size, (ii) Bartlett’s Test of Sphericity, and (iii) communalities, were taken. The Kaiser–Meyer–Olkin (KMO) sampling adequacy test was conducted for both individual items and constructed factors, and was deemed adequate if the value was greater than 0.6 [17–19] or given a maximum sample-item ratio of 20:1 [20]. A Bartlett’s Test of Sphericity was also used to measure the overall significance of correlations among all items in the measuring instrument. In other words, one can determine whether or not the original correlation matrix is an identity matrix; a significant *P*-value of 0.05 indicates that the data do not produce an identity matrix, and thus there is a multivariate normal distribution in which EFA can be performed [21]. Communality is described as the proportion of common variance within an observed variable. A communality value of less than 0.3 indicates that the item does not fit with other items in its factor [19, 21].

The number of factors that should be retained in the model was determined using Kaiser’s criteria and Scree-plot. In addition, factors with Eigenvalues exceeding one and factors lying above the elbow of the scree-plot were retained [21]. Generally, an item load greater than 0.3 is acceptable [22, 23]. However, taking item cross-loading into account, items are more important when loadings are close to one, so we decided to set an item loading threshold of > = 0.5. After factor extraction, the factors’ interpretation was made by factor rotation. There are two types of rotation: oblique and orthogonal; choosing the type of rotation depends on the goal of the study. If the goal is to get results that are the “best fit of the data”, oblique rotation is the way to go [24]. We also assumed that extracted factors may not be uncorrelated to each other. Considering these, we applied the oblique promax type of rotation. A factor with weak loading (0.5) or cross-loading was not retained [17].

### Confirmatory factor analysis

Many of the assumptions in the EFA are also shared by the CFA. Since there were no previously constructed latent variables, we decided to apply EFA in order to identify the number of latent variables. Following the execution of the EFA, a measurement model was applied to examine CFA using three critical results: parameter estimates, fit indices, and modification indices. The factor loading was acceptable if it was above 0.5 [25]. The overall model fit was assessed by different model fit indices. We used root mean square error of approximation (RMSEA), where values between 0.05 and 0.08 were considered an adequate model fit. Comparative fit index (CFI) and Tucker-Lewis index (TLI) values of 0.95 indicated good model fit, and values of 0.90 to 0.95 indicated acceptable model fit. The chi-square test was also calculated but not used to assess the model fit because of its sensitivity to large sample size. For Standardized Root Mean Square Residual (SRMR), values were considered good when they were between 0.0–0.05 and acceptable for values between 0.5–1.0 [25–27].

### Reliability test

Scale reliability or internal consistency was estimated following EFA. The internal consistency was estimated via Cronbach’s alpha coefficient and was tested for factors that emerged from factor analysis, as well as for the attitude scale items as a whole. Internal consistency was acceptable when Cronbach’s alpha was > 0.7; a score > 0.90 was considered to be excellent [26, 28]. Composite reliability (CR) and average variance extracted (AVE) were applied following CFA to estimate the reliability of the constructed validity. Scores > 0.4 for AVE and > 0.6 for CR generally indicate that the constructed validity was reliable [28–30].

### Regression analysis

A composite score value was generated from the 28 items and was used as a dependent variable. Three predictors, of which one was a continuous-discrete variable (work experience) and the two others were categorical variables (exposure to HEP & qualification), were selected and their association was tested using analysis of variance (ANOVA). A clinician’s “exposure to HEP” was assessed using three dichotomous questions. These included their involvement in HEP review meetings, HEP outreach engagement, and home visits to implement HEP. Generally, a clinician was considered as having an exposure to HEP if he/she responded “Yes” to at least one of the above questions. A linear regression model was conducted and a model goodness-of-fit test was checked using adjusted R-square (r^2^). Multicollinearity was checked by variance inflated factor (VIF) and was said to be present if VIF scored > 10% or mean VIF > 3%. The significance level was reported at a *P*-value < 0.05.

### Ethical issues

Ethical approval was obtained from the Ethiopian Public Health Institution (EPHI) prior to conducting this study. Supporting letters were obtained from the Federal Ministry of Health (FMoH), respective Regional Health Bureaus (RHB), and the district health offices of the study sites. Participants were informed of the nature of the study and provided their informed verbal consent prior to being interviewed.

## Results

### Characteristics of respondents

As indicated in Table 1, a total of 1210 clinicians were interviewed, with a response rate of 97.7%. The mean age of respondents was 28.3 (5.4 SD) years. More than half (53.4%) of respondents were in the age group of 25–29 years. 615 (50.8%) and 515 (49.2%) were male and female respondents, respectively. More than half (51.8%) of respondents grew up in rural residences up to the age of 15 years. Over half of the clinicians were married (621, or 51.3%). Professionally, most respondents (42.8%) were clinical nurses. Half of the respondents (50.58%) were degree holders with an average number of service years of 5.3, with the majority (53.1%) having served less than 5 years. 941 clinicians (77.8%) were working in health centers, with more than half (51.5%) working in rural facilities.

**Table 1:** Socio-demographic characteristics of respondents and clinician attitude, Ethiopia, 2019

Demography Variables	Response	Frequency	Percentage
Age in complete years	20–24	239	19.75
	25–29	646	53.39
	30–34	184	15.21
	> = 35	141	11.65
Marital status	Single	580	47.9
	Married	621	51.3
	Other^a^	9	0.74
Profession	Medical Doctor	71	5.87
	Nurse	518	42.81
	Midwife	395	32.64
	HO	199	16.45
	ISO	27	2.23
Highest level of education	Diploma	543	44.88
	Degree	612	50.58
	Masters	23	1.9
	Specialist	32	2.64
Service years as clinician	1–4 years	642	53.06
	> = 5 years	568	46.94
Type of facility currently working in	Hospital	269	22.23
	Health center	941	77.77
Location of facility currently working in	Rural	623	51.5
	Urban	587	48.5

NB: service year was classified using the mean as a reference but age was categorized based on authors’ consensus

^a^represents those divorced, widowed, etc.

A composite score value was generated for the 28 items, giving a score of 0 to 112. The mean and median for the composite score value were 78 (14.7 SD) and 80, respectively. More than half (58%) of respondents had a composite score value greater than or equal to the mean.

### Exploratory factor analysis

A simple frequency table was generated to identify any missing values, but all items were responded to. The floor and ceiling effects were calculated and, as a result, the floor effect (0/112) was 0.08% whilst the ceiling effect (112/112) was 0.17%. For the 26 items, the median value reported was 3. The mean of the item scores ranged from 1.5 to 3.2, while 50% (14 items) had a score of three and above.

**Fig. 1:**
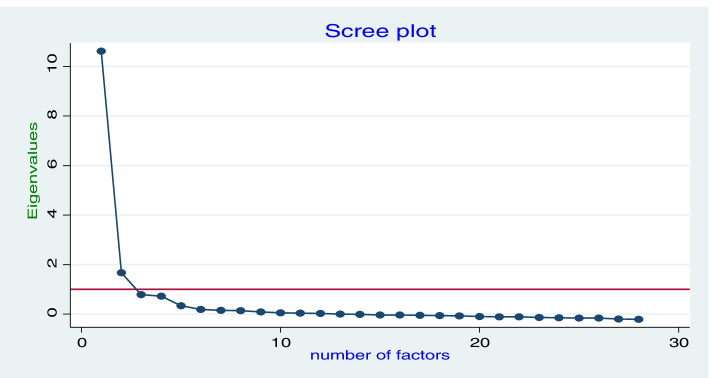
Scree plot used to detect the number of retained factors, Ethiopia, 2019

To ensure that the survey tool was suitable for EFA, statistical tests such as KMO, Bartlett’s test of sphericity, and communalities were applied. Sampling adequacy tested by KMO was reported at 0.96, ranging from 0.85 to 0.97, which falls under the marvelous category and indicates that items were sampled adequately. Bartlett’s test of sphericity was reported at chi^2^ (405) = 17,886.43 and *P* < 0.000. This rejects the ‘null hypothesis’ and accepts the alternative hypothesis, or factorability. The communality test was within an acceptable range of > 0.3 except for one item (AW02, communality = 0.27). Given all these confirmations using such statistical tests, exploratory factor analysis was conducted with the 28 items and, as illustrated in Fig. 1, the presence of two factors was revealed, which are labeled as: (1) clinician attitude towards the skill of HEWs (F1), and (2) clinician attitude towards the impact of HEP on community health improvement (F2). These two factors explain 93.2% of the total variance, with F1 and F2 accounting for 80.5 and 12.7% of the variances, respectively.

As seen in Table 2, 19 items with an item loading > = 0.5 were retained in the model, where 12 items were constructed for F1 and 7 items were constructed for F2. Sampling adequacy was calculated for the constructed factors and was reported at KMO = 94.6% (93 to 96%) for F1 and KMO = 92% (90 to 92.8%) for F2. Following factor extraction, a Promax oblique rotation was applied to identify the item structure for interpretation. Weakly loaded items (< 0.5) were excluded, which resulted in 19 of 28 items being retained, and no items were found to be cross-loaded. The percentage of variance was changed after the oblique rotation. F1 changed from 80.5 to 72.2% and F2 changed from 12.7 to 61.8%.

**Table 2:** EFA loading and Cronbach’s Alpha for clinician attitude, Ethiopia, 2019

Code	Question**	F1	F2
AW01	I believe that rural HEWs can efficiently conduct rapid diagnostic for malaria if they receive training.	0.67	
AW02	I am confident that rural HEWs can effectively provide community-based Tb DOTs	0.55	
AW03	I believe that rural HEWs can effectively treat under five children with diarrhea through training	0.77	
AW04	I believe that rural HEWs can effectively treat under five & adults with malaria if they got training	0.78	
AW05	With training, I believe that rural HEWs can effectively treat under five children with Pneumonia	076	
AW06	I believe that rural HEWs can effectively refer under five children with danger signs if they got training	0.60	
AW07	I believe that rural HEWs can treat under five children with ear problems if they got training	0.66	
AW08	I believe that rural HEWs with training can effectively treat under five children with malnutrition	0.62	
AW09	I am sure rural HEWs can effectively implement medical Abortion if this task is shift to them with training		
AW10	I believe that HEWs can contribute meaningful identification and referral of cases to higher level facility	0.57	
AW11	I believe that rural HEWs can provide long acting reversible contraceptive including IUCD if they got training	0.52	
AW12	I support and feel confident that rural HEWs can provide uncomplicated delivery service at health post level		
AW13	I accept and believe that rural HEWs can effectively deliver first aid services emergency cases at health post		
AW14	I accept and believe that rural HEWs can successfully provide vaccination for children and mothers	**0.58**	
AW15	I believe that Adolescent health needs such as behavioral change and family planning can be addressed through rural HEWs	**0.56**	
AW16	I believe that clinicians have to give value and respect for the works done by HEWs		
AW17	I would be happy to work with rural HEWs in any health related activity		
AW18	I do not have doubt on the competence of rural HEW to run their daily activities		
AW19	I believe that rural HEWs are well trained and qualified to the level their job demands		
AW20	I believe that rural HEWs are playing their role in improving community health needs		
AW21	Generally I support the existence and continuity of rural HEWs activity in the community		
AP01	I believe that rural HEP has been promoting community health needs well		**0.73**
AP02	I believe that rural HEP has been meeting Health care needs of hard to reach communities		**0.67**
AP03	I thought that health seeking behavior of rural community has increased after the implementation of Rural HEP		**0.77**
AP04	I thought that rural HEP has contributed to decreased maternal and under five mortality in rural community		**0.79**
AP05	I believe that rural HEP is a necessary and desirable for improvement of community health needs		**0.76**
AP06	In my view, primary healthcare delivery coverage is improved since the implementation of Rural HEP		**0.68**
AP07	Overall, rural HEP has a significant impact on improvement of community health in rural Ethiopia		**0.75**
	Cronbach’s alpha	0.91	**0.90**

** Items were rated on a 0 to 4 scale with 0 being ‘strongly disagree’ and 4 being ‘strongly agree’. Loadings < 0.50 are omitted

### Confirmatory factor analysis

**Fig. 2:**
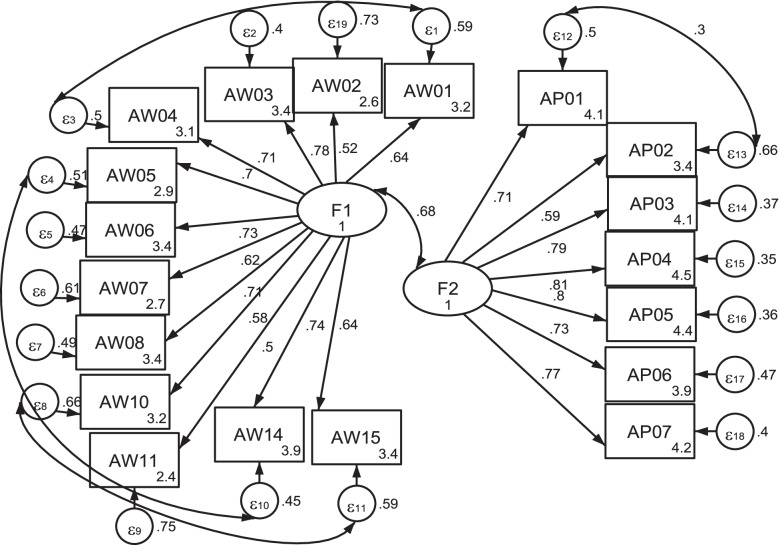
Standardized confirmatory factor analysis result, Ethiopia, 2019

The EFA finding revealed the presence of two latent factors which contributed to the clinician’s attitude towards HEP. We conducted CFA in order to verify the factorial validity of clinician attitude and to generate evidence regarding the fitness of the suggested model in relation to the structure of the factors identified via EFA. As illustrated in Fig. 2, results of the CFA show that the factor loading or path coefficient was > 0.5, ranging from 0.58 to 0.81. All items were significantly correlated with *P* < 0.00. The overall model fitness was acceptable, where chi^2^ was reported at (151) =1029.5, *P* < 0.00, RMSEA = 0.069, CFI = 0.928, TLI = 0.919, and SRMR = 0.042. However, some observed variables from the same latent factor showed high correlate error terms. To solve this problem, we generated a modification index to determine how much the model’s goodness of fit can be improved if variables with high error terms were correlated. A modification was made between AW01 & AW04, AW05 & AW14, AW10 & AW15, and AW14 & AW15 for the latent variable of F1 and also between AP01 & AP02 for the latent variable of F2. The modified model provides a better fitting index. Except for chi^2^, the other model fit indices fell under the “perfect fit criterion” category: RMSEA = 0.04, CFI = 0.97, TLI = 0.97 and SRMR = 0.03.

### Reliability test

Average inter-item variance and covariance were examined and reported at 0.26 and 0.36, respectively. The correlation of an item in relation to the total scores of all the other items was calculated to determine the correlation of a given item with the overall scale, where a correlation of < 0.20 was considered poor. However, the 28 items had an item-total correlation score ranging from 0.22 to0.70, indicating good correlation. The Cronbach’s α value for the 28 items was computed at 0.94. In order to see if Cronbach’s α would change substantially, the model was re-tested for items with a good item-total correlation score (> 0.3) by reversing Aw09 (0.22) and Aw12 (0.30), which had a relatively lower item-total score correlation. Reversing the two items resulted in reducing Cranach’s α from 0.94 to 0.93, thus ensuring a good statistical distribution of the data around the mean. Furthermore, Cronbach’s α was examined for items retained in each of the two latent variables and was determined to be 0.91 for F1 and 0.90 for F2. The use of composite reliability (CR) and average variance extracted (AVE) has a pivotal role in assessing factorial validity. In this study, the AVE for F1 and F2 was reported at 0.46 and 0.56, respectively. Similarly, the CR for F1 was 0.48 and 0.57 for F2.

### Regression analysis

Multiple regression analyses were carried out for the composite score value (dependent variable) and sample characteristics (independent variables). The relationship between the composite score value of the clinician attitude questionnaire and three independent variables (exposure to HEP, level of education, and work experience) were assessed. The three predictors showed significant correlation (*P* < 0.25) in the analysis of variance. However, only two predictors (exposure to HEP and level of education) were significant in the multiple linear regressions, controlling for confounders. This study shows that clinicians with exposure to HEP had a higher attitude score by 3.5, 95% CI (1.5, 5.3), and with a *P*-value < 0.001 than non-exposed. However, level of education had a negative correlation with clinician attitude, with degree-holding clinicians having a lower attitude score by − 2.7, 95% CI (− 4.4, − 0.94), and with a *P*-value < 0.002 than diploma-holding clinicians. There was no multi-collinearity as proven by VIF, where the mean was reported at 1.1%.

## Discussion

Even though clinicians are expected to engage in different activities of HEP, their attitudes towards the program have not been assessed previously. The HEP is not a temporary program; rather, it is an integral part of the Ethiopian health system [31] and its continuity is not in question. This study was therefore conducted to determine the reliability and validity of a clinician attitude measurement tool for future assessment of the program. This study also aimed to assess the correlation of selected predictors on clinician attitude. Four research questions were answered by these findings. First, we determined the number of latent variables by conducting an exploratory factor analysis. Second, we identified the level of reliability for general items and latent variables. Third, the validity of the research tool was assessed in a stepwise procedure. Last, two critical predictors of clinician attitude were identified through the regression model.

The EFA gave us two latent variables which explained 93.2% of the total variance. The identified latent variables were interpreted based on the loading factors that provided similar constructs. The first factor was interpreted as the attitude of clinicians towards HEW’s skill. Attitude toward the impact of HEP on community health improvement emerged as a second latent construct; this is useful for scholars and stakeholders in the health system who are unsure whether or not the HEP has had an impact. Other evaluative research also raises these points as important areas in the assessment of HEP [2, 4, 32]. These two latent variables are meaningful and relate to the empirical dimension of HEP. As clearly stated in the roadmap of Ethiopian HEP, [33] HEW’s skills and the impact of the program are the main priority areas that the Federal Ministry of Health plans to emphasize over the coming 15 years. Even though the absence of previous similar studies makes comparability difficult, other variances reported by non-similar work were close to this finding and were also considered a good indicator of model fit [17, 34]. However, this variance was nearly three times higher than a scale measure in the USA (variance = 32%) [35].

One of the objectives of this study was to ensure the validity of the clinician assessment tool. The CFA indicated that the scale had acceptable model fit indexes for RMSEA, SRMR, CFI, and LTI, but it was unfitted for chi^2^ exceptionally. After we applied model modification due to the error term, all the model fit indexes changed from the “acceptable” to the “perfectly fit criterion” category. However, chi^2^ remained unchanged. This finding leads us to the conclusion that the CFA affirmed the suitability of this questionnaire for measuring clinician attitude using two perspectives: HEW’s skill and the impact of HEP. A reliability test was done for factorial validity purposes using CR and AVE for both constructed sub-scales. Overall, the validity analyses showed that all the factor loading, CR, and AVE values were > 0.50, which indicates the presence of reliability.

Internal consistency is commonly estimated by Cronbach’s alpha coefficient [36] expressed as a number between zero and one, where the closer to one, the better the model fit [37, 38]. Overall, internal consistency for the 28 items was reported at 0.94. Measurement error, defined as one minus the square of the Cronbach’s alpha coefficient, was calculated at 11%, indicating that items in the instrument were correlated with each other. However, this is not guaranteed as the number of items in the test would also increase the alpha coefficient. The literature suggests that sampling adequacy is considered to be good if at least ten subjects per item are sampled in total [39]; taking this into account, a total of 280 samples would have been required in this study. 1210 samples were included, which is more than four times the required number. Therefore, the high reliability may be attributable to the high sample size inclusion. Reliability for sub-scale was examined after factor loading was conducted, and internal consistency was adequate for all factors.

Generally, as the level of education increased, the clinician attitude score decreased; on average clinician attitude decreased by − 2.7 for degree-holders compared with those who are diploma-holders. As confirmed in the descriptive statistics, the majority of degree and above holders work in hospitals where exposure to HEP is not common, whereas diploma-holders are usually assigned to HCs. According to the current Ethiopian health system structure, HPs are directly linked with catchment HCs for administrative as well as support reasons. Therefore, the discrimination in the predictive values of the different components may relate to the level of involvement. On average, clinician attitude increased by 3.5 for those who had exposure to HEP compared to those that did not have exposure.

## Conclusion

The EFA revealed the presence of two latent variables. Meanwhile, the assessment tool has demonstrated high reliability as confirmed by Cronbach’s alpha, composite reliability, and average variance extracted. The confirmatory factor analysis affirmed that this tool was valid to measure clinician attitudes towards HEP. Clinician attitude has a direct and positive relationship with exposure to HEP. We recommend that clinicians need to have continuous exposure to the HEP program in order to enhance their attitude.

## Data Availability

The corresponding author will provide data, upon request.

## Abbreviations

AA: Addis Ababa
ANOVA: Analysis Of Variance
AVE: Average Variance Extracted
CAF: Confirmatory Factor Analysis
CI: Confidence Interval
CIF: Comparative Fit Index
CR: Composite Reliability
DD: Dire Dawa
EFA: Exploratory Factor Analysis
FMoH: Federal Ministry of Health
GH: General Hospital
GP: General Practitioner
HC: Health Center
HEP: Health Extension Program
HEW: Health Extension Worker
HO: Health Officer
HP: Health Post
ISO: Integrated Surgical Obstetrician
KMO: Kaiser Meyer-Olkin
MD: Medical Doctor
ODK: Open Data Kit
PHCU: Primary Health Care Unit
RH: Referral Hospital
RMSEA: Root Mean Square Error Administration
SRMR: Standardized Root Mean Square Residuals
TLI: Tuckler-Lewis Index
VIF: Variance Inflated Factor
